# Nutritional, health and environmental dimensions of Swiss food consumption trends

**DOI:** 10.3389/fnut.2025.1677951

**Published:** 2025-11-18

**Authors:** Alba Reguant-Closa, Daria Loginova, Eric Mehner, Stefan Mann, Thomas Nemecek

**Affiliations:** 1 Life Cycle Assessment Research Group, Agroscope, Zürich, Switzerland; 2 Department of Socioeconomics, Agroscope, Ettenhausen, Switzerland

**Keywords:** food consumption, environment, health, trend analysis, nutrition, dietary recommendations

## Abstract

**Introduction:**

Food has a considerable environmental impact beyond its effects on the nutritional and health status of the population. Food consumption changes over time and is influenced by diverse socioeconomic factors. This study aimed to evaluate the nutritional, health, and environmental (NHE) dimensions of foods commonly consumed by the Swiss population, assess consumption trends in combination with the NHE dimensions from 1990 to 2017 at the food and diet levels, and suggest recommendations for consumption pattern improvement.

**Methods:**

The nutrient rich food index 10.3 (NRF10.3) was used to evaluate the nutritional dimension, while the health nutritional index (HENI) evaluated the health effects of dietary intake. The environmental dimension of the foods was assessed by LCA using the SALCA method v2.0.1. To evaluate consumption trends, we used data on Swiss household-level purchases provided by the Swiss Federal Statistical Office for the years 1990, 2000, 2010, and 2017. Using regression analysis, we estimated the trends of NHE dimensions combined with consumption at the productand household diet levels.

**Results:**

The analysis showed many trades-offs between the dimensions. At the food group level, the decreased consumption of all meat except poultry had a positive nutritional and health impact while decreasing theoverall environmental impact of meats. At the diet level, an increase in nutritional density was observed over time, while the HENI index was higher in 2000. The environmental dimension was highly dependent on which impact category was observed.

**Discussion:**

Three main recommendations can be drawn from this study: First, it is important to include several dimensions in food and diet analysis. Second, decreasing red and processed meat and increase of poultry consumption through the years can maintain the nutrient density of the diets, improve its health impacts, and decrease the majority of the environmental impact categories compared to diets with high red meat consumption. Third, the consumption of pulses, fruits, vegetables, and nuts was very low compared to the recommendations, but increasing it can improve all the dimensions studied.

## 1 Introduction

Food production and consumption have a considerable environmental impact (1, 2), with a clear influence of consumption on the nutritional and health status of the population (3, 4). Several studies support the idea that certain dietary patterns can reduce the risk of non-communicable diseases and increase the provision of critical micronutrients (5–7). However, food preferences are highly influenced by socioeconomic factors and changes over time (8). Understanding the dynamics of the nutritional, health, and environmental (NHE) dimensions of food consumption is of high importance for reducing its negative impacts on the environment and increasing the nutritional and health status of a population.

Generally, food-based dietary guidelines (FBDGs) (9) have been shown to be well aligned with sustainability principles for NHE dimensions (10), although in some cases, a more restricted modification of the guidelines to limit the consumption of high environmental impact foods has been suggested (11, 12). However, many studies have shown that people do not follow FBDGs, albeit there is some convergence between eating and guidelines over time (13). Instead, most populations demonstrate various eating patterns influenced by socioeconomic factors, intuition, beliefs, and nutritional knowledge (14, 15). Additionally, FBDGs are only updated every few years, while the food industry is a thriving and dynamic sector, rapidly innovating and releasing products, some of which have yet to be included in FBDGs. This is the case, for example, with plant-based food alternatives, which have been on the rise in recent years (16) but still have not been incorporated in the majority of FDBGs.

Several countries use household consumption data to understand consumption behavior and develop consumption-based policies that reconcile the various dimensions of food production and consumption (17, 18). The Swiss household consumption survey allows for analyzing randomly selected households in Switzerland over 27 years (1990–2017) and has previously been used to demonstrate significant trend changes between foods, as well as a detailed analysis with inter- and pan-generational differences in food consumption (15). However, the associated studies did not include NHE analyses of Swiss food consumption.

The case of Switzerland is of particular interest because it allows for studying the NHE dimensions of food consumption with various high-quality sources of data and with large datasets already available for food consumption by the Swiss population. For example, the menuCH survey (19) showed that people in Switzerland do not follow FBDGs, recording higher intakes of meat, alcohol, and processed foods and lower amounts of fruits, vegetables, and pulses than recommended. Consequently, household consumption and consumption trend studies can help reveal synergies and trade-offs between NHE dimensions to align food consumption with sustainability goals (20). We expect that since consumption changes over time, the NHE dimensions will also show variations, which could inform valuable recommendations on food consumption from an NHE perspective. Revealing trends over time can support developing recommendations that support sustainable consumption at the household and national levels, that is, at the level of policies and general health and environmental recommendations. Our findings contribute to the identification of potential synergies and trade-offs in dietary trends over the studied years and help to decrease the environmental impact and improve the nutrition and health of the Swiss population in the future.

Despite the high interest in the topic and its importance for both nature and society, our study, to the best of our knowledge, is the first to assess the NHE dimensions of food consumption patterns in Switzerland from 1990 to 2017. The aim of this study is threefold: to evaluate the NHE dimensions of foods commonly consumed by the Swiss population; to assess consumption trends in combination with the NHE dimensions from 1990 to 2017 at the product and diet levels; and to suggest recommendations for consumers and policy makers to improve consumption patterns in the Swiss population aligned with the three studied dimensions (NHE).

## 2 Data and methods

This study uses three main data sources: (1) Swiss household food purchase data, which was used to assess consumption at the household level; (2) the Swiss food composition database, which was used to define the nutritional content of the foods included in the analysis; and (3) life cycle inventories, which were used to assess the environmental impact of the foods. The subsections below provide details of the approaches we used to retrieve and merge information from the databases for our analyses. Table 1 shows the main characteristics of the data we used.

**Table 1:** Summary of the data sources.

Dataset	Data type	Data selection
Consumption	Household consumption data collected over 1 month at 1990, 2000, 2010, and 2017	Average monthly purchases of 77 different food items in grams per person, measured and stored each year in FSO (21)
Nutrition	Nutrient composition	Swiss food composition database (25)
Health	Nutrient composition	Swiss food composition database (25)
	Dietary risk factors (DRF)	Swiss DRF based on data from the global burden of disease study based on Ernstoff et al. (35)
Environment	Life cycle inventories	Original and modified life cycle inventories from ecoinvent, SALCA, WFLDB and Agribalyse
Food waste	Household food waste	Adjusted by each food group based on data by Beretta et al. (46)

### 2.1 Household food purchase data

We used disaggregated household data on Swiss household consumption provided by the Swiss Federal Statistical Office for the years 1990, 2000, 2010, and 2017 for 6–12 thousand randomly selected survey participants (households of Switzerland) each year (21). The data collected comprised a random sample of households in Switzerland who reported their personal characteristics and kept food purchasing diaries. Therefore, the data we used were the result of a reliable randomized observational survey.

The list of personal characteristics, survey frequency, and food classifications varied over time, and we performed all necessary matching to ensure comparability of the data for the years 1990, 2000, 2010, and 2017. The average amounts of consumption (purchases) per person calculated for this database were similar to official statistics (22) but smaller than declared by some Swiss non-governmental organizations, because the latter use food balances and add restaurant food and foods consumed by tourists to their averages for the population. A main shortcoming of our data is the lack of consideration of shifts from home to out-of-home food consumption. To account for this shortcoming, we considered the share of restaurant food 15% of the total food consumption (19, 23). In addition, the weight declared by households is usually the weight of food before preparation (cooking, pealing), and containing the weight of some bones for meat. This shortcoming might be meaningful [for example for potato weight (24)]. To account for this limitation, we considered food waste and losses at the consumer stage (see Section 2.4), for example, by considering food preparation residuals such as peels. However, we did not account for household cooking methods. While we acknowledge that omitting the cooking stage introduces limitations, we opted for this approach to avoid introducing additional variability and uncertainty relating to variety of household cooking methods which have different impacts on the nutritional content of foods as well on its environmental impacts.

Nevertheless, the database we used is meaningful for the analysis conducted in this study because it is the biggest and most reliable available data source for consumption in Switzerland.

In the absence of data for a particular year and food category, an average of the other years’ data entries was used as a proxy for the calculations. For example, when consumption of pulses for the year 2017 was not available, the average of the years 1990, 2000, and 2010 was used. Our outlier policy involved the exclusion of 0.5% of households with minimum and maximum consumption per food and year to obtain robust estimates.

### 2.2 Nutritional data

To calculate the health and nutritional indices, the Swiss Food Composition Database (SFCDB) was selected to provide the nutritional composition of foods (25). The SFCDB database contains 1,059 food items based on Swiss food consumption and cuisine customs and thus is a good representation of Swiss food composition. Food categories from the purchasing household data were matched to nutritional entries of the SFCDB to best represent consumption patterns. A matching of all the entries is detailed in Supplementary Table S2 with the appropriate references. The main adaptations are described below.

- When a food category average was available in the SFCDB, it was applied (e.g., “beef meat (average)”).
- When possible, food categories were divided into different food items based on menuCH data (19) to better represent Swiss food consumption. This was applicable when food categories were defined only in general terms in the household food purchase data (i.e., fish), and no detail of the category was given (i.e., fatty vs. non-fat fish), which could significantly impact the results of the nutritional and health dimensions. Even though menuCH was recorded only during the years 2014–2015, this is currently the best nutritional survey at the country level in Switzerland. Therefore, the same percentages and weightings of food items were applied for all the years considered in this study.
- This study did not consider cooking methods. Nutritional and health indices were calculated based the weights of reported rawfoods. This may have a considerable impact on some foods that gain weight when cooked. For example, cooking pasta will double its weight, but its nutritional content will half. Thus, the trend of the nutritional and health index will remain the same while some values may change slightly. Exceptions were made when a processing step was specified (e.g., canning and drying), in which case it was considered for the calculation of the nutritional and health indices.
- When data were not available on the composition of food categories, and menuCH did not provide the information, we used other reliable sources (all detailed in Supplementary information). For example, consumption for sweetened versus non-sweetened yogurts was not specified on menuCH, and thus, other sources were used (26).
- When data were not available on the composition of food categories (e.g., types of fruit for the category canned fruit) and menuCH or any other source did not provide the information, but many foods were available on the SFCDB, an average of the available foods was included.

There were 14 food groups for which the foods included were not specified or too generic (see Supplementary Table S5). Hence, they were considered “unspecified” food groups. For the diet-level analysis, the nutritional value of the unspecified food groups were extrapolated from the total food consumed. The proportion of the unknown categories is 4.9, 4.0, 5.9, and 6.8% for the years 1990, 2000, 2010, and 2017, respectively. Consequently, only 63 food groups were included in the food group analysis.

#### 2.2.1 Nutritional dimension

To evaluate the nutrient density of food products, an adaptation of the Nutrient Rich Food Index (NRF) 9.3 (27, 28) was proposed for this study: NRF10.3. This modified NRF index consists of three sub-indices: (1) the nutrient-rich index (NR), including 10 qualifying nutrients; (2) the limiting nutrient index (LIM), including three disqualifying nutrients; and (3) NRF10.3, which is the difference between the former two (see Table 2).

**Table 2:** Formula for the Nutrient Rich Food Index (NRF) 10.3.

Indices	Nutrients considered (reference intake per person and day)	Formula	Scale
NR10	Protein (61 g) Dietary fiber (30 g) Vitamin A (700 μg RE) Vitamin C (102.5 mg) Vitamin E (12 mg ATE) Ca (100 mg) Fe (13.5 mg) Mg (325 mg) K (3,500 mg) I (150 μg)	$\mathrm{NR}=\sum (\frac{\mathrm{QN}}{\mathrm{DRI}})$	Larger values show better dietary quality
LIM3	Saturated fats (25 g) Total sugar (56 g) Na (2000 mg)	$\mathrm{LIM}=\sum (\frac{\mathrm{DN}}{\mathrm{MRV}})$	Larger values show worse dietary quality
NRF10.3	NR and LIM nutrients	NRF10.3=(NR−LIM)	Larger values show better dietary quality

Source: Adapted from Fulgoni and Keast (28). Units: dimensionless. NR10, nutrient rich index; RE, retinol equivalents; ATE, alpha-tocopherol; QN, amounts of qualifying nutrients on the food analyzed; DRI, dietary reference intakes; LIM3, limiting nutrient index; DN, amounts of disqualifying nutrients on the food analyzed; MRV, maximum reference values; NRF10.3, Nutrient-Rich Food Index 10.3. Values in parentheses are reference values corresponding to the DRI and MRV (see Supplementary Table S1).

In contrast to NRF9.3, iodine was included as a qualifying nutrient, as its deficiency is a well-known issue in the Swiss population (29, 30). Given that this is a Swiss case study, dietary reference intake (DRI) and maximum reference values (MRV) are considered for the Swiss adult population (see Supplementary Table S1).

To calculate NRF10.3, we made several important assumptions:

- A DRI average for men and women was used (see Supplementary Table S1).
- For sugars, only “free sugars” were considered, following the WHO (31) recommendation of no more than 10% of daily energy intake.
- For sweets and soft drinks, we assumed that the total sugar content was added and thus counted as free sugars.
- For processed fruit, such as jams, total sugar content was also considered total free-sugar content due to its processing and high glycemic index (as details on how much sugar comes from fruit and how much from added sugars were lacking).
- For sweetened products, the free-sugar content was calculated by subtracting the total sugar content of the unsweetened products from that of the sweetened product (e.g., natural yogurt vs. sweetened yogurt).

**Figure 1:**
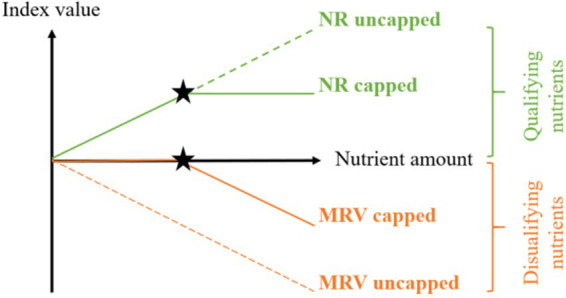
Schematic representation of the capping (diet level) and uncapping (food group level) systems for qualifying (green) and disqualifying (red) nutrients. * Represents the capping points for qualifying (100% of DRI) and disqualifying (100% of MRV) nutrients.

For the analysis at the product level (per 100 g), capping was not applied, as high contents of one nutrient in a food can compensate for low contents in another food (32, 33). However, for the analysis at the diet level (g of food consumed/person/day), capping was applied to qualify nutrients at the maximum level of the DRI (because higher levels of qualifying nutrients do not imply a higher nutritional value in a balanced diet). For disqualifying nutrients at the diet level, nutrient contents were considered only when they exceeded the MRV values (see Figure 1).

**Figure 2:**
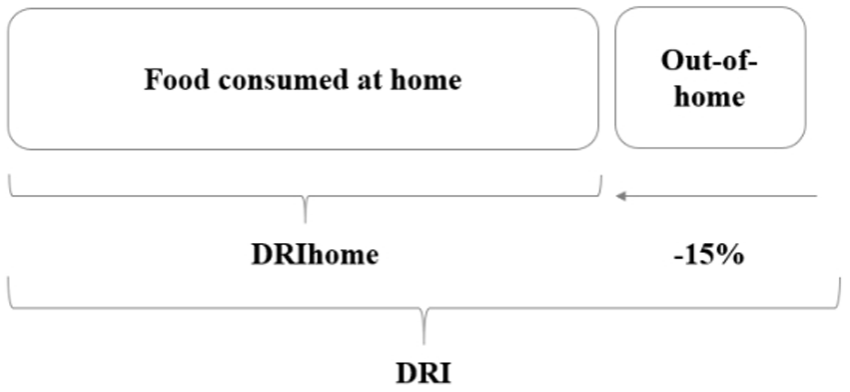
Methodological adaptation from DRI to DRIhome.

To compare the results at the diet level, the DRI values were adapted to include out-of-home consumption. The purchasing data at the household level omits the food consumed out of home. In this study, our estimates showed that 15% of the food was consumed outside the house for the Swiss population (19, 23). Thus, for the diet-level comparison, the dietary reference intakes (DRI) were reduced by 15%, accounting only for the food consumed at home to ensure unbiased comparison with the nutritional recommendations. The adjusted reference values were named DRIhome (Figure 2).

#### 2.2.2 Health data and dimension

The health dimension was evaluated using the Health Nutritional Index (HENI), as described by Stylianou et al. (34). Swiss dietary risk factors from the global burden of disease study were used based on data by Ernstoff et al. (35). The HENI analysis included 15 dietary risk factors and alcohol consumption (see Table 3). Trans-fatty acids (TFA) were not considered, as their content was not available in the SFCDB database. Trans fatty acid contents in foods have been a health concern in Switzerland in the past, especially in processed foods (36), but large efforts have been undertaken to regulate and reduce their contents (37). Thus, only small differences in the results would be expected if TFA were considered. Stylianou et al. (34) also found that TFA had a relatively small impact on HENI scores for most of the food categories (34). Omega-3 fatty acids were not included in the SFCDB, but as they have considerable relevance to the HENI score (34), and fatty fish is one of the main fishes consumed in Switzerland (19), omega-3 values for seafood (DHA and EPA values) were complemented from the USDA food composition database (38). The HENI score was calculated at the product (per 100 g of food product consumed) and diet (per gram of food consumed/person/day) levels for the 4 years considered in this study. Since we did not consider the cooking stage to avoid adding variability on the data, the HENI index was calculated based on raw products for those bought raw (see Supplementary Table S2 for details of the reference products used for the calculations).

**Table 3:** Formula for the Heath Nutritional Index (HENI).

Dietary risk factors	Formula	Scale
Calcium, dietary fiber (other), PUFA, fiber (f, v, l, w), sodium, nuts and seeds, whole grains, legumes, fruits, vegetables, milk, processed meat, red meat, omega-3 (from seafood), SSB, alcohol	HENIp=−0.53∗(∑DRFr∗ap,r)	Negative values indicate its consumption increases the risk of detrimental health effects

Source: adapted from Stylianou et al. (34); Units: μDALY. f, fruits; v, vegetables; l, legumes; w, whole grains; p, food product; ap,r, amount of dietary risk component *r* in food item *p*, DRF_r_, cumulative age and gender adjusted marginal DRF per g of dietary risk *r* in μDALYs g ^−1^; PUFA, polyunsaturated fatty acids; SSB, sugar sweetened beverages.

### 2.3 Environmental dimension

#### 2.3.1 Goal and scope definition

The environmental assessment of all food groups considered was conducted via a life cycle assessment (LCA) according to international standards (60). The goal was to assess the environmental impact of the food groups considered in this study at the product (per 100 g) and diet (per grams of food consumed/person/day) levels. The system boundaries were set from agricultural production up to the processing gate or arrival in Switzerland for imported goods. Hence, retailer activities and food preparation were not included. Packaging and cooling/storing at the retailer generally have a relatively low share of the total impacts, so the results of this study would not have been substantially changed. For imported food products, the environmental effects of transport to Switzerland were considered separately for each country of origin.

#### 2.3.2 Life cycle inventory

Life cycle inventories were sourced from various databases, including SALCA, ecoinvent, Agribalyse, Agri-Footprint, and WFLDB. For each food group, import and export inventories were considered and weighted for their proportional contributions based on import/export data (39). For Swiss production, raw products were modeled based on internal data (40) and Swiss-specific ecoinvent inventories. If no inventory for Swiss production was directly available, the most suitable inventory from Agribalyse or ecoinvent was selected, and when possible, background processes were regionalized to suit Swiss production practices. For the imported products, all available inventories in the databases Agribalyse, Agri-Footprint, ecoinvent, SALCA, and WFLDB were considered. Import mixes were created based on import data (39), matching the available inventories with the observed countries of origin. Food processing inventories were based on the best available inventories, which were adapted to the regional conditions of the respective country of origin for both imported and domestically produced food. If the above-described approach was not viable for a food product, the best available proxy was selected. The same inventory data were used for all the years considered. Details of all inventories selected for each food category can be found in the Supplementary Table S3.

#### 2.3.3 Life cycle impact assessment

The life cycle impact assessment was performed using the software SimaPro v.9.5.0 (61), applying the SALCA v.2.0.1 methodology (41). For the results section, seven ICs were selected for discussion in detail, as they are the most commonly applied ICs in food LCAs (1). First, we included global warming (GW) with a 100-year time horizon, as proposed by the Intergovernmental Panel on Climate Change (42). Second, water scarcity based on the available water remaining (AWARE) model (43) was included, estimating the potential of water deprivation in a specific watershed. Next, we provided terrestrial acidification, freshwater eutrophication, and marine eutrophication, all of which are based on the model proposed by ReCiPe 2016 v1.1 (Hierarchist) (44). Freshwater eutrophication is mainly driven by phosphorus emissions, while marine eutrophication is mainly driven by nitrogen emissions. Thus, both ICs represent different impacts and were included in the analysis. Land use was considered in the shape of agricultural land occupation based on the ReCiPe Midpoint Hierarchist v2008 method (62). Finally, freshwater ecotoxicity (45) was included as the sum of the organic and inorganic emissions of toxic chemicals to freshwater ecosystems.

### 2.4 Food waste

To account only for consumed foods in the nutritional analysis, the average waste rates per food category at the household level were calculated. As specific data on household food waste were not available for each of the 4 years considered in this study, food waste proportions were kept constant based on data by Beretta et al. (46). Nutritional and health indices were calculated based on the estimated food consumed. By contrast, the environmental impacts of foods were calculated considering the full amount purchased without subtracting food waste, as it contributed considerably to the final environmental impact of the food basket.

### 2.5 Identification of NHE indices and calculating trends in scores

The final list of indices used at the product and diet levels in this study is as follows:

**Table:** 

Nutrition	Health	Environment
NRF10.3 NR10 LIM3	HENI	Water scarcity: AWARE (WS) Land occupation: Agricultural (LO) Global warming: 100 years (GW) Eutrophication: Marine (EM) Eutrophication: Freshwater (EF) Acidification: Terrestrial (AT) Ecotoxicity: freshwater USEtox (ET)

To understand the dynamics of the NHE indices at the food group level, we multiplied the values of the studied indices (per 100 g) by the consumption amounts (in 100 g) in the different years. We called these resulting values “scores.” The names of the scores are equivalent to the names of the NHE indices. They change yearly in accordance with consumption and numerically represent the respective indices. We defined the vectors of the scores as p for the food group level and as d for the diet level.

We aimed to identify significant trends in the scores p for 63 studied food groups (each denoted as i ), and trends in the scores d including all 77 food categories. As we had 11 NHE indices, we needed to calculate 63 × 11 score trends at the food group level and 11 score trends at the diet level. To do so, We used robust linear regressions for each score p at the food group level (Equation 1a) and 11 more regressions for revealing trends in scores d at a dietary level (Equation 1b) both over time t but obtaining tseparate estimates for trends $\beta_{i}$ and φ :


$$p_{i,t}=\alpha_{i}+\beta_{i}t+\varepsilon_{i,t},t\in [1990,2000,2010,2017]$$ (1a)

$$d_{t}=\gamma +\varphi \;t+\xi_{t},t\in [1990,2000,2010,2017]$$ (1b)

Where $\alpha_{i,}$ and γ are constants, and $\varepsilon_{i,t}\;$ and $\xi_{t}$ are the error terms. Therefore, the trends in our study were measured by regressing the volume of the score of interest on a time variable.

Given that 11 indices were included in this analysis, particular caution is required in interpreting both the terminology and the outcomes. As with consumption patterns—where an upward trend may be considered beneficial (e.g., in the case of vegetables) or detrimental (e.g., in the case of salt and sugar)—a positive dynamic in the indices does not invariably denote improvement. For example, a decline in the environmental index reflects a reduced impact of consumption on the environment, and thus constitutes an improvement. Conversely, declines in the nutritional and health indices imply less nutritious and less healthy diets, and therefore represent a deterioration. The trends in all indices are zero because the indices do not change over time; however, trends in consumption differ between foods. Multiplying consumption on the stable coefficients of NHE dimensions changes the slopes of the trends.

## 3 Results

The results present the NHE dimensions of consumption trends over the period of 1990–2017 by food group, aggregating food groups by the levels of the Swiss pyramid and at the household diet level.

### 3.1 NHE dimensions of food consumption trends by food group

We measured trends for each combination of the studied indices and food items, providing insights into the development of the impact of Swiss consumption on NHE. Nine food groups had a significantly positive and twenty-three a significantly negative yearly consumption trend measured in average monthly grams per person. However, when combined with the NHE dimensions, food categories did not behave the same for all dimensions, showing synergies and trade-offs. This allowed for investigating the impacts of Swiss purchasing patterns on NHE dimensions. Low-nutrient-density foods, such as fruits and vegetables, had a low nutritional density according to the NRF10.3 but a positive health impact based on the HENI index. Although meats had good nutrient density, they generally ranked worse on the HENI index (especially red and processed meats). Furthermore, foods high in sugar (such as sweets or pastry) ranked worse in NRF10.3 than in HENI. However, some foods had similar behaviors in all dimensions (e.g., beans and milk). See Supplementary Table S4 for the nutritional and health indices of all food groups per 100 g.

**Figure 3:**
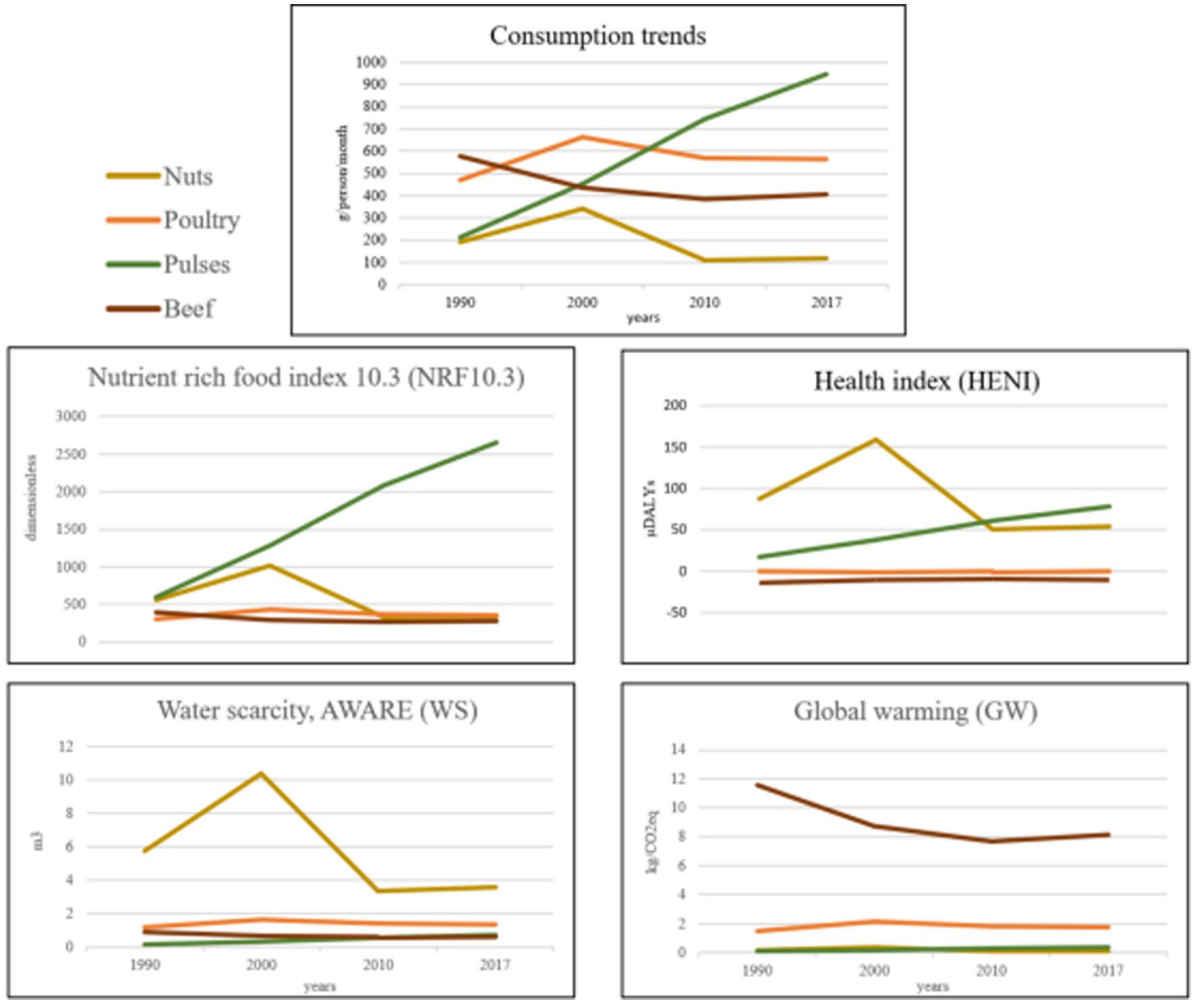
The graphs compare nuts, poultry, pulses, and beef consumption trends from 1990 to 2017. The Consumption Trends graph shows pulses increasing while others fluctuate. The rise in pulses consumption, increase The Nutrient Rich Food Index. The rise in nuts consumption in the year 2000 increase The Health Index and The Water Scarcity. With the decrease in beef consumption, Global Warming decreases.

Figure 3 shows an example of the consumption trends and the NHE dimensions of four selected foods. The pulse consumption trend increased, showing a positive effect on nutrition and health while having a low environmental impact in all studied ICs. Thus, it had a positive effect on the health of the population as well as the planet. By contrast, the consumption of nuts decreased. Given their high nutrient density, a decrease in nut consumption over time negatively affected the nutrient intake of the population. Similarly, nuts had a high positive dietary risk on the HENI index; therefore, a decrease in their consumption had a negative impact on the health of the population. Concerning the environmental ICs, for water scarcity, nuts had a large environmental impact, and therefore reducing their consumption notably lowered their impact.

Other environmental ICs were not as strongly affected. Two types of meat were compared in this example, both of which behaved differently in each NHE dimension. Beef consumption decreased, while poultry consumption increased. Environmentally, this switch in meat consumption generally had a positive effect with a clear decrease in the GW. The health dimension also benefited from this change in consumption, as red meat ranked negatively on the HENI index, while poultry had an almost neutral effect. At the nutritional level, both meats had a similar index, and the switch did not lead to large differences in the final nutritional status of the population.

Table 4 shows the results for 18 food groups for which the estimates of trends in consumption were significant (*p* < 0.1). As for the other 59 of the 77 food groups, the scores did not change. The freshwater eutrophication (EF) and marine eutrophication (EM) indices were significant for most foods, but their magnitude was close to zero for all foods. Thus, we conclude that the effects of consumption change are relatively small. A similar situation was observed for acidification terrestrial (AT) dynamics. The highest magnitude was observed for some dairy products: cheese and curd (0.004), milk (−0.002), butter (−0.002), and cream (−0.001), as well as for dried vegetables and mushrooms (−0.002), cocoa and chocolate (0.001), and pork (−0.001). The positive sign for AT-score trends indicates that a change in consumption led to a reduction in the environmental impact.

**Table 4:** Trends in nutritional, health, and environmental (NHE) scores (only significant trends are shown).

Food	Consumption	ET	AT	EF	EM	GW	LO	WS	HENI	NRF10.3
Apples	−6.55E+01 (2.05E+01)*	−5.38E+00 (1.68E+00)*	−4.00E-05 (1.00E-05)*	0 (0)*	−5.00E-05 (1.00E-05)*	−6.00E-03 (0)*	−1.90E-02 (1.00E-02)*	−1.50E-02 (0)*	−3.01E+00 (9.40E-01)*	−1.22E+01 (3.81E+00)*
Beans and peas	2.73E+01 (7.00E-01)***	3.04E+01 (7.80E-01)***	6.00E-05 (0)***	1.00E-05 (0)***	3.00E-05 (0)***	1.10E-02 (0)***	3.80E-02 (0)***	2.00E-02 (0)***	1.90E+00 (5.00E-02)***	6.45E+01 (1.66E+00)***
Bread	−2.78E+01 (5.46E+00)**	1.05E+01 (2.06E+00)**	−1.30E-04 (3.00E-05)**	0 (0)**	−5.00E-05 (1.00E-05)**	−1.40E-02 (0)**	−3.20E-02 (1.00E-02)**	−4.00E-03 (0)**	−7.64E-01 (1.50E-01)**	−7.52E+00 (1.48E+00)**
Butter	−1.25E+01 (2.41E+00)**	−3.80E+00 (7.30E-01)**	−1.50E-03 (2.90E-04)**	−1.00E-05 (0)**	−1.40E-04 (3.00E-05)**	−1.35E-01 (3.00E-02)**	−1.48E-01 (3.00E-02)**	−6.00E-03 (0)**	−8.30E-02 (2.00E-02)**	7.78E+00 (1.50E+00)**
Cheese and curd	4.90E+01 (9.53E+00)**	5.68E+01 (1.11E+01)**	4.22E-03 (8.20E-04)**	5.00E-05 (1.00E-05)**	4.20E-04 (8.00E-05)**	4.16E-01 (8.00E-02)**	4.39E-01 (9.00E-02)**	3.80E-02 (1.00E-02)**	−3.65E-01 (7.00E-02)**	3.23E+01 (6.30E+00)**
Cocoa and chocolate	2.26E+01 (7.46E+00)*	6.56E+00 (2.16E+00)*	5.70E-04 (1.90E-04)*	3.00E-05 (1.00E-05)*	2.10E-04 (7.00E-05)*	3.28E-01 (1.10E-01)*	1.72E-01 (6.00E-02)*	4.00E-02 (1.00E-02)*	2.90E-01 (1.00E-01)*	−1.11E+01 (3.64E+00)*
Cream	−1.53E+01 (5.19E+00)*	−1.99E+00 (6.70E-01)*	−7.60E-04 (2.60E-04)*	−1.00E-05 (0)*	−7.00E-05 (2.00E-05)*	−7.00E-02 (2.00E-02)*	−7.40E-02 (2.00E-02)*	−1.00E-03 (0)*	−2.40E-02 (1.00E-02)*	−1.07E+00 (3.60E-010.36)*
Dried vegetables and mushrooms	2.21E+01 (5.81E+00)*	3.69E+02 (9.70E+01)*	1.62E-03 (4.30E-04)*	6.00E-05 (1.00E-05)*	4.00E-05 (1.00E-05)*	4.27E-01 (1.10E-01)*	2.00E-02 (1.00E-02)*	4.98E-01 (1.30E-01)*	−1.51E+00 (4.00E-01)*	6.37E+01 (1.67E+01)*
Egg	−2.12E+00 (5.00E-01)*	−3.20E-01 (7.60E-02)*	−9.00E-05 (2.00E-05)*	0 (0)**	−1.00E-05 (0)*	−3.00E-03 (0)*	−6.00E-03 (0)*	−1.00E-03 (0)*	5.00E-03 (0)*	−1.90E+00 (4.50E-01)*
Leafy vegetables	−3.34E+01 (1.13E+01)*	−5.93E+01 (2.01E+01)*	−1.70E-04 (6.00E-05)*	−1.00E-05 (0)*	−2.00E-05 (1.00E-05)*	−3.80E-02 (1.00E-02)*	−1.00E-02 (0)*	−3.00E-03 (0)*	−7.14E-01 (2.40E-01)*	−2.69E+01 (9.11E+00)*
Lemons	4.05E+01 (8.08E+00)**	3.27E+01 (6.52E+00)**	1.30E-04 (3.00E-05)**	1.00E-05 (0)**	1.00E-05 (0)**	1.30E-02 (0)**	1.60E-02 (0)**	2.49E-01 (5.00E-02)**	2.24E+00 (4.50E-01)**	3.72E+01 (7.42E+00)**
Margarine	−6.53E+00 (1.01E+00)**	−1.33E+00 (2.10E-01)**	−1.20E-04 (2.00E-05)**	0 (0)**	−3.00E-05 (1.00E-05)**	−1.40E-02 (0)**	−4.30E-02 (1.00E-02)**	−2.50E-02 (0)**	−1.36E-01 (2.00E-02)**	−5.94E-01 (9.00E-02)**
Milk	−1.81E+02 (3.85E+01)**	−5.56E+00 (1.18E+00)**	−2.37E-03 (5.10E-04)**	−2.00E-05 (0)**	−2.30E-04 (5.00E-05)**	−2.09E-01 (4.00E-02)**	−2.36E-01 (5.00E-02)**	−1.20E-02 (0)**	−2.15E-01 (5.00E-02)**	−5.85E+01 (1.25E+01)**
Olive oil	6.57E+00 (1.55E+00)*	2.76E+01 (6.53E+00)*	1.50E-04 (3.00E-05)*	1.00E-05 (0)*	2.00E-05 (0)*	1.00E-02 (0)*	4.70E-02 (1.00E-02)*	5.00E-02 (1.00E-02)*	7.30E-02 (2.00E-02)*	1.26E+01 (2.99E+00)*
Onions and Garlic	3.67E+01 (5.92E+00)**	9.96E+00 (1.61E+00)**	9.00E-05 (1.00E-05)**	0 (0)**	2.00E-05 (0)**	6.00E-03 (0)**	1.00E-02 (0)**	1.30E-02 (0)**	7.81E-01 (1.30E-01)**	8.91E+00 (1.44E+00)**
Pears and quinces	2.53E+01 (1.95E+001.95)***	2.14E+00 (1.70E-01)***	2.00E-05 (0)***	0 (0)***	2.00E-05 (0)***	2.00E-03 (0)***	9.00E-03 (0)***	5.00E-03 (0)***	1.41E+00 (1.10E-01)***	6.53E+00 (5.00E-01)***
Pork	−7.17E+00 (1.99E+00)*	−1.35E+01 (3.73E+00)*	−7.00E-04 (1.90E-04)*	0 (0)*	−9.00E-05 (3.00E-05)*	−4.40E-02 (1.00E-02)*	−4.80E-02 (1.00E-02)*	−9.00E-03 (0)*	1.64E-01 (5.00E-02)*	−3.76E+00 (1.04E+00)*
Sugar	−1.96E+01 (5.82E+00)*	−1.09E+01 (3.23E+00)*	−1.40E-04 (−4.00E-05)*	0 (0)*	−3.00E-05 (1.00E-05)*	−8.00E-03 (0)*	−1.30E-02 (0)*	−1.00E-03 (0)*	0 (0)*	4.04E+01 (1.20E+01)*

Significance codes: ***p ≤ 0.01; **p ≤ 0.05; *p ≤ 0.1; Values in brackets are standard errors. Food classification by Mann and Loginova (15). ET, Ecotoxicity wP USEtox freshwater; AT, Acidification terrestrial; EF, Eutrophication freshwater; EM, Eutrophication marine; GW, Global warming; LO, Land occupation, agricultural; WS, Water scarcity, AWARE; HENI, Heath Nutritional Index; NRF10.3, Nutrient Rich Food Index 10.3.

The decrease in consumption of apples, leafy vegetables, bread, cream, margarine, and milk results in a decrease in ecotoxicity freshwater (ETF), global warming potential (GWP), land occupation (LO), water scarcity (AWARE), health impacts (HENI), and nutritional density (NRF10.3). In general, these values suggest a decrease in nutritional and health dimensions but a lower environmental impact. An opposite trend was observed for beans and peas, lemons, olive oil, onions and garlic, and pears and quinces, whose consumption increased, with all scored trends being positive, suggesting improvement in nutritional and health dimensions but increased environmental impacts.

For the last group of foods, the nutritional and health dimensions exhibited a dynamic trend opposite to consumption. A decrease in butter and sugar consumption resulted in a negative trend for all indices but positive dynamics of NRF10.3. A decrease in pork and egg consumption resulted in positive dynamics of HENI (0.16 and 0.005, respectively), while other indices decreased. Positive dynamics of consumption for cheese and curd, dried vegetables, and mushrooms led to negative HENI dynamics (−0.36 and −1.5, respectively). A similar observation was recorded for cocoa and chocolate, resulting in a negative NRF10.3 value (−11.05).

### 3.2 NHE dimensions of consumption trends by aggregating food groups by the levels of the Swiss food pyramid

**Figure 4:**
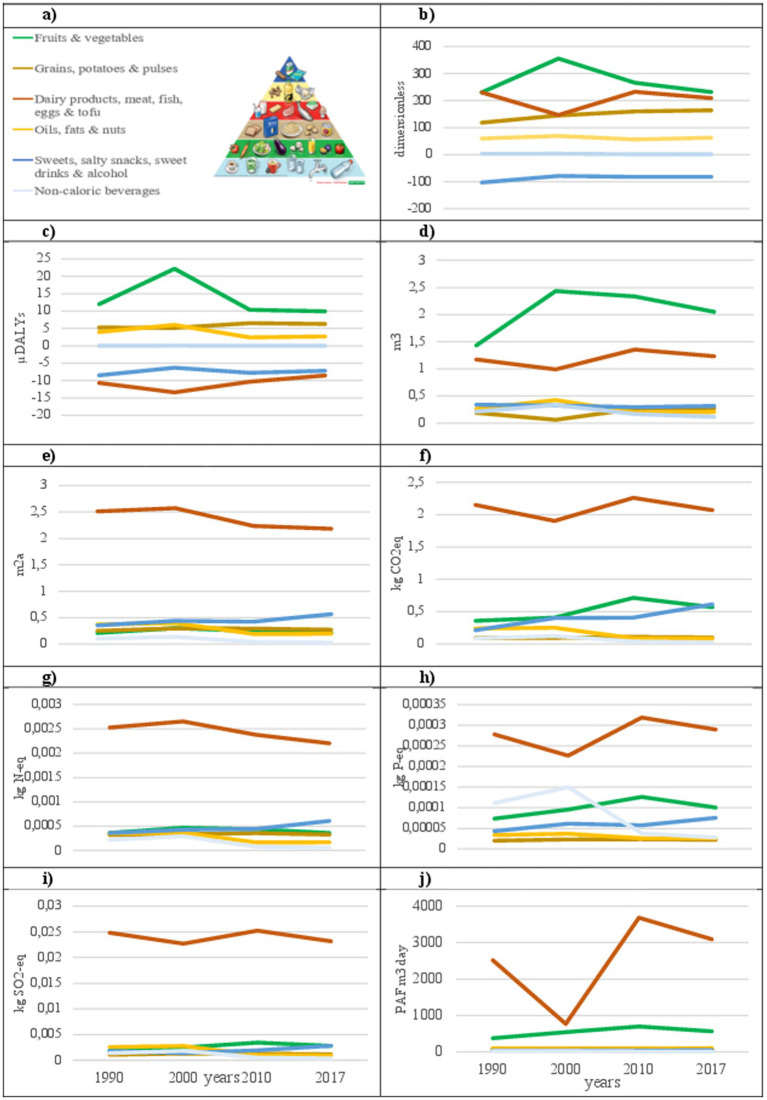
These graphs illustrate various food categories following the Swiss food pyramid labels. Line charts show trends from 1990 to 2017 in various metrics such as nutrient density, microDALYs, water usage, carbon emissions, and other environmental impacts, each color-coded line to match the food pyramid’s categories. These charts depict changes over time across the categories.

For this analysis, food groups were aggregated following the six levels defined by the Swiss food pyramid (SFP): (1) non-caloric beverages (water, tea, coffee); (2) vegetables and fruits; (3) grains, potatoes, and pulses; (4) dairy, meat, fish, egg, and tofu; (5) oils, fats, and nuts; and (6) sweets, salty snacks, sweet drinks, and alcohol. Figure 4 shows foods per grams consumed per person and day according to the levels of the SFP (63).

Due to their low nutrient density, the contribution of non-caloric beverages to nutritional and health impacts was close to zero. Environmental impacts were considerable for WS, EM, EF, and AT, which were mainly driven by coffee. Fruits and vegetables had the second-highest NRF10.3 and the highest HENI values of all SFP levels. Environmentally, they had a low impact, except for WS and ET, which showed the highest and second highest impacts at all levels, respectively. The third level of the SFP had the highest NRF10.3 as well as a high HENI value, mostly driven by the consumption of pulses. Additionally, grains, potatoes, and pulses had a low environmental impact in all ICs analyzed. Level 4 of the SFP had a good NRF10.3 value but a negative HENI score due to red and processed meat consumption. Across the included years, there was a decrease in the consumption of milk, yogurt, and red and processed meat but an increase in poultry consumption. This group had the highest environmental impact for all ICs except WS. The environmental impact of each IC changed over the years, depending on the share of foods consumed that year. As this level of the SFP contains a wide range of foods that differ considerably in the NHE dimensions, a detailed analysis of the group is provided in Supplementary Figure S1.

The oils, fats, and nuts levels had moderate NRF10.3 and HENI scores. The HENI score was highest in 2000, mainly due to high nut consumption. The nutrient density of this category is moderate to low, with few modifications over time. Environmentally, it has a moderate impact for all categories, with a peak in the year 2000 for some ICs, especially WS, also influenced by increased nut consumption. The top level of the SFP had a negative HENI and NRF10.3 index while having a relatively moderate environmental impact, which increased over the years due to the increased consumption of sweet, salty snacks and sweetened and alcoholic drinks (Figure 4).

### 3.3 NHE dimensions of consumption trends at the diet level

To represent aggregated food consumption at the household level, we performed an analysis at the diet level by aggregating the consumption of all foods per year, person, and day (Table 5). As described in the methodology, the diet-level analysis in this study considered only household consumption, not out-of-home consumed food. At the diet level, the largest differences in NHE dimensions were observed for the HENI scores, which were significantly higher in 2000 due to the share of foods consumed in that year (mainly driven by the increased consumption of nuts, vegetables, fruits, and fatty fish).

**Table 5:** NHE dimensions of consumption trends at the diet level from 1990 to 2017.

	1990	2000	2010	2017
NRF10.3	9.23E+00	9.50E+00	9.98E+00	1.00E+01
HENI	2.11E+00	1.42E+01	1.57E+00	3.25E+00
WS	3.60E+00	4.73E+00	4.75E+00	4.31E+00
LO	3.98E+00	4.38E+00	3.62E+00	3.64E+00
GW	3.33E+00	3.38E+00	3.83E+00	3.67E+00
EM	4.36E-03	4.82E-03	4.06E-03	3.94E-03
EF	5.93E-04	6.29E-04	6.28E-04	5.76E-04
AT	3.52E-02	3.42E-02	3.52E-02	3.29E-02
ET	3.37E+03	1.69E+03	4.96E+03	4.18E+03

NRF10.3, Nutrient Rich Food Index 10.3; HENI, Heath Nutritional Index; WS, Water scarcity, AWARE; LO, Land occupation agricultural; GW, Global warming; EM, Eutrophication marine; EF, Eutrophication freshwater; AT, Acidification terrestrial; ET, Ecotoxicity freshwater. Green indicates better impacts, orange shows medium impacts, and red indicates the worst impacts.

At the diet level, NRF10.3 was the only index with a significant trend estimate (*p* ≤ 0.05). The nutrient density of the diet increased from year to year due to an increase in the NR values as well as an increase in the LIM values. The LIM values were mainly influenced by increased consumption of sweets, pastry, and sweetened drinks. The environmental impact at the diet level fluctuated between the years, and the individual IC were highly dependent on the proportions of food consumed each year, which hindered observing distinct trends. Although many environmental ICs of the Swiss diets have improved over the last decades, GW and ETF showed a tendency to increase. We also observed that the year 2017 ranked lowest for most of the analyzed ICs.

## 4 Discussion

This study investigated the NHE dimensions of foods commonly consumed by the Swiss population. Whereas earlier studies have explored the environmental impacts and dietary quality of Swiss diets, to our knowledge, this study is the first to analyze the NHE dimensions of Swiss household consumption data from 1990 to 2017.

### 4.1 Evaluation of NHE dimensions at the food group level

We found that the NHE dimensions of the food groups analyzed changed over time, showing many trade-offs between the different dimensions studied. Despite the link between the health and nutrition dimensions—that is, good nutrition leads to better health—this study shows that the relationship is not *sine qua non*, and a more nutritious diet is not always healthier. The chosen indices for nutrition (NRF10.3) and health (HENI) for this study seemed to behave differently for the same foods. For example, NRF10.3 considers sugars a limiting nutrient, ranking the food group of sweets and pastry the lowest. By contrast, the HENI index considers only sweetened sugar beverages as a dietary risk factor but not the sugar content of other foods, even as added sugar. Thus, both groups ranked much better on the HENI index. However, the HENI demonstrated a high sensitivity to some nutrients or food groups with large positive (e.g., nuts) or negative (e.g., processed meats) dietary risk factors (4, 34). The same was true for the environmental ICs, which had large variability depending on the food share of each food group and year. For example, Level 4 of the SFP (dairy, meat, fish, egg, and tofu) showed a higher environmental impact for almost all ICs, especially red and processed meat. However, for other food groups (e.g., nuts, fruits, and vegetables), Level 4 ranked low in almost all ICs except WS, showing clear trade-off between ICs. We also observed variability among each food group and for the three dimensions, depending on consumption amounts and food share changes over time.

### 4.2 Evaluation of NHE dimensions at the diet level

Our analysis at the diet level shows a higher HENI score for the year 2000. The HENI index considers nuts, whole grains, fruits, vegetables, and high omega-3 content from seafood as positive dietary risk factors, which were all highly consumed that year, raising the index value. Some authors have argued that HENI results have to be interpreted and used with caution due to differences in the relevance of the food groups and nutrients considered by this index (64). Nevertheless, we believe that using this index in combination with NRF10.3 deepens the analysis of food consumption, which incorporates the health impacts of dietary patterns and not only their nutrient density (47, 48).

At the nutritional level, an overall increase in nutrient density was observed across the included years. However, both the NR and LIM sub-indices increased, leading to a higher intake of saturated fats, sugar, and sodium. The increase in the LIM index was mainly driven by a rise in the consumption of canned food (high in sodium), sweetened beverages, pastry, and sweets. We also acknowledge that this study did not analyze all the dimensions of diet quality (e.g., nutrient quality or diversity), which are vast (49, 50). However, many authors have reported the difficulty of including all aspects of nutrition in a single indicator (51, 52). To better tackle the nutritional dimension of the target population, a modified NRF index was selected to include iodine, which is commonly deficient in the Swiss population (29, 30). Additionally, population-adjusted DRI was established (as an average for men and women based on Swiss DRI; see Supplementary Table S1).

Nevertheless, caution should be exercised when interpreting our results, as new deficiencies may arise due to new food tendencies in the future (53). For example, switching to more plant-based nutrition could lead to deficiencies in other nutrients, such as vitamin B12 or calcium. Thus, the inclusion of other nutrients in the NRF could be considered in the future to evaluate suitable nutrient adequacy aligned with new consumer trends. Finally, the environmental dimension behaves differently depending on food and IC, and it was not possible to derive significant trends. Nevertheless, trade-offs and synergies between food groups, ICs, and years were observed (LO, EM, EF, and AT showed a tendency to decrease, while GW and ETF were lower in the first studied years), highlighting the importance of evaluating more than one IC to avoid burden shifting.

Some of the food consumption behaviors observed in this study are aligned with other nutritional studies carried out in the Swiss population. The Swiss national survey, menuCH, was carried out on a sample of 2057 participants during 2014–2015 using two 24-h recalls across the three main language-speaking regions of Switzerland (54). Krieger et al. (55) identified four dietary patterns: (1) Swiss traditional (high intake of chocolate and dairy products); (2) Western 1 (high intake of soft drinks and meat); (3) Western 2 (high intake of alcohol, meat, and starchy); and (4) prudent (high intake of fruits, vegetables, white meat, and fish). These results align with our consumer data, which show high intakes of alcoholic drinks, sweet beverages, sweets, and chocolate. The consumption of these foods has also been associated with an increase in non-communicable diseases observed in the Swiss population (56) and in other developed countries (57). In our study, meat consumption decreased for all meats (beef, veal, pork, sheep, goat, and horse meat, and hare and wild meat) except poultry. For the different years, the sum of all meats was 94 g/person/day (year 1990), 108 g/person/day (year 2000), 82 g/person/day (year 2010), and 74 g/person/day (year 2017), which is higher than what is recommended per day on the SFP (47 g/person/day) but lower than what was reported in the menuCH study (124 g/person/day).

We have indicated that our study did not include out-of-home consumption, which was included in the menuCH data. The observed change in meat consumption in our study did not affect nutrient density or improve the health dimension and most environmental ICs. In fact, reducing meat consumption is one of the main strategies for reducing dietary environmental impacts (58). Some authors have advocated for a strict reduction of meat intake to improve the environmental impact of the population (11, 12). However, when meat is substituted with plant-based alternatives, special attention must be paid to the nutritional adequacy of the overall diet, as the nutritional content of plant-based products can differ from that of animal-based products (53, 59). The household food purchase data used for our study reported a high consumption of canned foods, especially fish and vegetables. The consumption of these foods is not reported in menuCH or other surveys carried out in the Swiss population, and therefore cannot be verified. Hence, considering their high consumption (up to 75 g/person/day for canned fish in 2017), caution is warranted in drawing conclusions on the NHE impacts of these food groups.

### 4.3 Limitations

In this study, we aimed to accurately match household purchase data with nutrient content and environmental inventories for each food category. However, some uncertainties had to be assumed from this matching, as food groups were in some cases broad (e.g., *confiserie*) and at other times very specific (e.g., beef meat) (see Supplementary Tables S2, S3). When the food selection for a category had a significant influence on the final results, we focused on including the best data available to reflect the Swiss food market and consumer preferences. For a more detailed food group distribution, menuCH was used, as it reflects real-life consumption data. For example, it was used to divide the group “fish” by fish type (e.g., salmon, tuna, etc.), as in this case, the share of each fish type will have a significant impact on the environment. When specific data were not available in menuCH, other databases were used. Yogurt, for example, can be sweetened or unsweetened, which affects NRF10.3. Thus, Swiss consumption data on yogurt preferences were used to reflect this share (26).

Additionally, the nutrient content of foods might change over the years due to novel product developments or new food regulations (e.g., it was recently found that the sodium content of bread and the sugar content of sweetened products decrease over time). As these changes are very difficult to reflect, we did not consider them. Accordingly, we assumed the food nutrient content remained stable over the years. For the environmental inventories, we did not consider modifications to agricultural management strategies, emission changes, or import/export dynamics through the years. However, we considered the Swiss food system diversity in the food inventories (see Supplementary Table S3). For example, each food group was divided into an import and a domestic production share. For imports, each country of origin and type of agricultural production was considered when available, including transportation to Switzerland. In the case of Swiss domestic production, we differentiated between mountain, hill, and plain regions, as well as production systems (e.g., organic vs. conventional or open field vs. greenhouse production), when possible.

## 5 Conclusion

This study provided a combined analysis of consumption trends with the NHE dimensions of foods. The study aimed to analyze consumption trend effects in three dimensions: nutrition, health, and environment. Household Swiss population data from 1990 to 2017 were analyzed at the food group and household diet levels. From the results of this study, three main recommendations can be drawn. First, it is important to evaluate the various dimensions of food when analyzing population consumer trends to identify synergies and trade-offs between the different dimensions. Second, the replacement of red and processed meat with other meats, such as poultry, can maintain the nutrient density of the diets, improve its health impacts, and decrease the majority of the environmental ICs compared to higher red and processed meat consumption. However, this study did not analyze the case of overall meat reduction, which was previously shown to reduce the environmental impact of diets, and which should be included in further research, especially when substituting with plant-based diets to prevent nutrient deficiencies. Third, as reported by others in previous literature, the consumption of pulses, fruits, vegetables, and nuts was very low compared to the recommendations, but its increase led to an improvement in all dimensions studied, especially as shown by the analysis of data from the year 2000. Thus, more agricultural and nutritional initiatives, as well as policy measures, should focus on promoting the consumption of these food groups.

## Data Availability

The original contributions presented in the study are included in the article/Supplementary material, further inquiries can be directed to the corresponding author.

## References

[1] Poore J Nemecek T. Reducing food’s environmental impacts through producers and consumers. Science. (2018) 360:987–92. doi: 10.1126/science.aaq0216 29853680

[2] Tilman D Clark M. Global diets link environmental sustainability and human health. Nature. (2014) 515:518–22. doi: 10.1038/nature13959, PMID: 25383533

[3] Krebs-Smith SM Pannucci TE Subar AF Kirkpatrick SI Lerman JL Tooze JA . Update of the healthy eating index: HEI-2015. J Acad Nutr Diet. (2018) 118:1591–602. doi: 10.1016/j.jand.2018.05.021, PMID: 30146071 PMC6719291

[4] Murray CJL Aravkin AY Zheng P Abbafati C Abbas KM Abbasi-Kangevari L . Global burden of 87 risk factors in 204 countries and territories, 1990–2019: a systematic analysis for the global burden of disease study 2019. Lancet. (2020) 396:1223–49. doi: 10.1016/s0140-6736(20)30752-2 33069327 PMC7566194

[5] Fresán U Sabaté J. Vegetarian diets: planetary health and its alignment with human health. Adv Nutr. (2019) 10:S380–s388. doi: 10.1093/advances/nmz019, PMID: 31728487 PMC6855976

[6] Henney AE Gillespie CS Alam U Hydes TJ Boyland E Cuthbertson DJ. Ultra-processed food and non-communicable diseases in the United Kingdom: a narrative review and thematic synthesis of literature. Obes Rev. (2024) 25:e13682. doi: 10.1111/obr.13682 38204299

[7] Paris JMG Falkenberg T Nothlings U Heinzel C Borgemeister C Escobar N. Changing dietary patterns is necessary to improve the sustainability of Western diets from a one health perspective. Sci Total Environ. (2022) 811:151437. doi: 10.1016/j.scitotenv.2021.151437, PMID: 34748829

[8] Frehner A Zanten HHEV Schader C Boer IJMD Pestoni G Rohrmann S . How food choices link sociodemographic and lifestyle factors with sustainability impacts. J Clean Prod. (2021) 300:126896. doi: 10.1016/j.jclepro.2021.126896

[9] FAO. (2024). Dietary guidelines. Available online at: https://www.fao.org/nutrition/education/food-dietary-guidelines/home/en/ (Accessed 01 December, 2024).

[10] Reynolds CJ Buckley JD Weinstein P Boland J. Are the dietary guidelines for meat, fat, fruit and vegetable consumption appropriate for environmental sustainability? A review of the literature. Nutrients. (2014) 6:2251–65. doi: 10.3390/nu6062251, PMID: 24926526 PMC4073148

[11] Frehner A Cardinaals RPM de Boer IJM Muller A Schader C van Selm B . The compatibility of circularity and national dietary recommendations for animal products in five European countries: a modelling analysis on nutritional feasibility, climate impact, and land use. Lancet Planet Health. (2022) 6:e475–83. doi: 10.1016/s2542-5196(22)00119-x, PMID: 35709805

[12] Willett W Rockström J Loken B Springmann M Lang T Vermeulen S . Food in the Anthropocene: the EAT-lancet commission on healthy diets from sustainable food systems. Lancet. (2019) 393:447–92. doi: 10.1016/s0140-6736(18)31788-4, PMID: 30660336

[13] Schneid Schuh D Guessous I Gaspoz J-M Theler J-M Marques-Vidal P. Twenty-four-year trends and determinants of change in compliance with Swiss dietary guidelines. Eur J Clin Nutr. (2019) 73:859–68. doi: 10.1038/s41430-018-0273-0, PMID: 30116035

[14] Loginova D Mann S. Sweet home or battle of the sexes: who dominates food purchasing decisions? Humanit Soc Sci Commun. (2024) 11:261. doi: 10.1057/s41599-024-02745-8

[15] Mann S Loginova D. Distinguishing inter- and pangenerational food trends. Agric Food Econ. (2023) 11:10. doi: 10.1186/s40100-023-00252-z

[16] Fehér A Gazdecki M Véha M Szakály M Szakály Z. A comprehensive review of the benefits of and the barriers to the switch to a plant-based diet. Sustainability. (2020) 12:4136. doi: 10.3390/su12104136

[17] Damari Y Kissinger M. Changing food preferences and choices – a framework for analyzing households food purchases over time. Int J Gastron Food Sci. (2024) 36:100920. doi: 10.1016/j.ijgfs.2024.100920

[18] Eyles H Dodd S Garton KK Jiang Y Gontijo de Castro T. New Zealand household purchases of sugar-sweetened, artificially sweetened, and unsweetened beverages: 2015–2019. Public Health Nutr. (2024) 27:e22. doi: 10.1017/S1368980023002793, PMID: 38115219 PMC10830360

[19] Chatelan A Beer-Borst S Randriamiharisoa A Pasquier J Blanco JM Siegenthaler S . Major differences in diet across three linguistic regions of Switzerland: results from the first national nutrition survey menuCH. Nutrients. (2017) 9:1163. doi: 10.3390/nu9111163 29068399 PMC5707635

[20] UN. (2024). United Nations. Available online at: https://sdgs.un.org/goals (Accessed 01 December, 2024)

[21] FSO (2024). Household budget survey. Available online at: https://www.bfs.admin.ch/bfs/en/home/statistics/economic-social-situation-population/surveys/hbs.html (Accessed November 4, 2022).

[22] SBV (2023). Statistische Erhebungen und Schätzungen über Landwirtschaft und Ernährung. Switzerland: Schweizer Bauernverband, Agristat, Brugg.

[23] Beretta C Hellweg S. Potential environmental benefits from food waste prevention in the food service sector. Resour Conserv Recycl. (2019) 147:169–78. doi: 10.1016/j.resconrec.2019.03.023

[24] Willersinn C Mack G Mouron P Keiser A Siegrist M. Quantity and quality of food losses along the Swiss potato supply chain: stepwise investigation and the influence of quality standards on losses. Waste Manag. (2015) 46:120–32. doi: 10.1016/j.wasman.2015.08.033 26341828

[25] FSVO. (2023). The Swiss food composition database. Available online at: https://naehrwertdaten.ch/en/#:~:text=The%20Swiss%20Food%20Composition%20Database%20is%20a%20data%20collection%20of,that%20are%20available%20in%20Switzerland.&text=The%20Swiss%20Food%20Composition%20Database%20contains%20information%20on%20the%20composition,that%20are%20available%20in%20Switzerland (Accessed July 3, 2022).

[26] Chatelan A Gaillard P Kruseman M Keller A. Total, added, and free sugar consumption and adherence to guidelines in Switzerland: results from the first national nutrition survey menuCH. Nutrients. (2019) 11:1117. doi: 10.3390/nu11051117 31109151 PMC6566881

[27] Drewnowski A Amanquah D Gavin-Smith B. Perspective: how to develop nutrient profiling models intended for global use: a manual. Adv Nutr. (2021) 12:609–20. doi: 10.1093/advances/nmab018, PMID: 33724302 PMC8166553

[28] Fulgoni VL 3rd Keast DR Drewnowski A. Development and validation of the nutrient-rich foods index: a tool to measure nutritional quality of foods. J Nutr. (2009) 139:1549–54. doi: 10.3945/jn.108.101360, PMID: 19549759

[29] Fischer L Andersson M Braegger C Herter-Aeberli I. Iodine intake in the Swiss population 100 years after the introduction of iodised salt: a cross-sectional national study in children and pregnant women. Eur J Nutr. (2024) 63:573–87. doi: 10.1007/s00394-023-03287-6 38141138 PMC10899291

[30] Haldimann M Bochud M Burnier M Paccaud F Dudler V. Prevalence of iodine inadequacy in Switzerland assessed by the estimated average requirement cut-point method in relation to the impact of iodized salt. Public Health Nutr. (2015) 18:1333–42. doi: 10.1017/S1368980014002018 (Accessed September 20, 2023). 25231207 PMC10271515

[31] WHO (2015) Guideline: Sugars intake for adults and children. Geneva, Switzerland: World Health Organization, (Accessed December 10, 2023). 25905159

[32] FAO. (2021). Integration of environment and nutrition in life cycle assessment of food items: Opportunities and challenges. Rome, Italy: Food and Agriculture Organization, (Accessed March 4, 2023).

[33] Hallström E Davis J Woodhouse A Sonesson U. Using dietary quality scores to assess sustainability of food products and human diets: a systematic review. Ecol Indic. (2018) 93:219–30. doi: 10.1016/j.ecolind.2018.04.071 (Accessed September 20, 2021).

[34] Stylianou KS Fulgoni VL Jolliet O. Small targeted dietary changes can yield substantial gains for human health and the environment. Nat Food. (2021) 2:616–27. doi: 10.1038/s43016-021-00343-4 (Accessed February 10, 2022)., PMID: 37118177

[35] Ernstoff A Stylianou KS Sahakian M Godin L Dauriat A Humbert S . Towards win-win policies for healthy and sustainable diets in Switzerland. Nutrients. (2020) 12:2745. doi: 10.3390/nu12092745 (Accessed September 25, 2021)., PMID: 32916882 PMC7551606

[36] Richter EK Shawish KA Scheeder MRL Colombani PC. Trans fatty acid content of selected Swiss foods: the TransSwissPilot study. J Food Compos Anal. (2009) 22:479–84. doi: 10.1016/j.jfca.2009.01.007 (Accessed January 28, 2022).

[37] WHO (2015). Eliminating trans fats in Europe, A policy brief. Available online at https://iris.who.int/handle/10665/363877 (Accessed October 10, 2023).

[38] USDA (2018). USDA food composition databases. Available online at: https://ndb.nal.usda.gov/ndb/ (Accessed November 29, 2023).

[39] BAZG (2023). Datenbank Swiss-Impex. Bundesamt für Zoll und Grenzsicherheit. AVailable online at: https://www.gate.ezv.admin.ch/swissimpex/ (Accessed April 10, 2023).

[40] Nemecek T Roesch A Bystricky M Jeanneret P Lansche J Stüssi M . Swiss agricultural life cycle assessment: a method to assess the emissions and environmental impacts of agricultural systems and products. Int J Life Cycle Assess. (2023) 29:433–55. doi: 10.1007/s11367-023-02255-w

[41] Douziech M. Bystricky M. Furrer C. Gaillard G. Lansche J. Roesch A. . (2024). Recommended impact assessment method within Swiss agricultural life cycle assessment (SALCA): v2.01 (Agroscope science, issue. Agroscope. Available online at: https://ira.agroscope.ch/en-US/publication/56332 (Accessed June 20, 2024).

[42] IPCC (2021). Climate Change 2021: The Physical Science Basis. Contribution of Working Group I to the Sixth Assessment Report of the Intergovernmental Panel on Climate Change. Available online at: https://www.ipcc.ch/report/sixth-assessment-report-working-group-i/ (Accessed February 4, 2022).

[43] Boulay A-M Bare J Benini L Berger M Lathuillière MJ Manzardo A . The WULCA consensus characterization model for water scarcity footprints: assessing impacts of water consumption based on available water remaining (AWARE). Int J Life Cycle Assess. (2018) 23:368–78. doi: 10.1007/s11367-017-1333-8

[44] Life Cycle Initiative (2022). Global guidance for life cycle impact assessment indicators and methods (GLAM) UN Environment Programme. Available online at: https://www.lifecycleinitiative.org/activities/key-programme-areas/life-cycle-knowledge-consensus-and-platform/global-guidance-for-life-cycle-impact-assessment-indicators-and-methods-glam/ (Accessed February 1, 2023).

[45] USEtox. (2019). USEtox (corrective release 2.12). Available online at: https://usetox.org/model/download/usetox2.12 (Accessed 02 February, 2024).

[46] Beretta C Stucki M Hellweg S. Environmental impacts and hotspots of food losses: value chain analysis of Swiss food consumption. Environ Sci Technol. (2017) 51:11165–73. doi: 10.1021/acs.est.6b06179, PMID: 28862841

[47] Guo A Bryngelsson S Strid A Bianchi M Winkvist A Hallström E. Choice of health metrics for combined health and environmental assessment of foods and diets: a systematic review of methods. J Clean Prod. (2022) 365:2622. doi: 10.1016/j.jclepro.2022.132622

[48] Jolliet O. Integrating dietary impacts in food life cycle assessment. Front Nutr. (2022) 9:898180. doi: 10.3389/fnut.2022.898180, PMID: 35911123 PMC9326460

[49] Fardet A Rock E. Toward a new philosophy of preventive nutrition: from a reductionist to a holistic paradigm to improve nutritional recommendations. Adv Nutr. (2014) 5:430–46. doi: 10.3945/an.114.006122, PMID: 25022992 PMC4085191

[50] Wirt A Collins CE. Diet quality - what is it and does it matter? Public Health Nutr. (2009) 12:2473–92. doi: 10.1017/S136898000900531X, PMID: 19335941

[51] Ocké MC. Evaluation of methodologies for assessing the overall diet: dietary quality scores and dietary pattern analysis. Proc Nutr Soc. (2013) 72:191–9. doi: 10.1017/S0029665113000013, PMID: 23360896

[52] Waijers PMCM Feskens EJM Ocké MC. A critical review of predefined diet quality scores. Br J Nutr. (2007) 97:219–31. doi: 10.1017/S0007114507250421, PMID: 17298689

[53] Mansilla R Long G Welham S Harvey J Lukinova E Nica-Avram G . Detecting iodine deficiency risks from dietary transitions using shopping data. Sci Rep. (2024) 14:1017. doi: 10.1038/s41598-023-50180-7 38200032 PMC10781720

[54] BLV. (2014). menuCH - Nationale Ernährungserhebung. Bundesamt für Lebensmittelsicherheit und Veterinärwesen (BLV). Available online at: https://www.blv.admin.ch/blv/de/home/lebensmittel-und-ernaehrung/ernaehrung/menuCH.html (Accessed July 2, 2022).

[55] Krieger JP Pestoni G Cabaset S Brombach C Sych J Schader C . Dietary patterns and their sociodemographic and lifestyle determinants in Switzerland: results from the national nutrition survey menuCH. Nutrients. (2019) 11:62. doi: 10.3390/nu11010062 PMC6356790 30597962

[56] Pestoni G Karavasiloglou N Braun J Krieger JP Sych JM Bopp M . Does diet map with mortality? Ecological association of dietary patterns with chronic disease mortality and its spatial dependence in Switzerland. Br J Nutr. (2022) 127:1037–49. doi: 10.1017/S0007114521001525 33971997 PMC8924527

[57] Bennett JE Stevens GA Mathers CD Bonita R Rehm J Kruk ME . NCD countdown 2030: worldwide trends in non-communicable disease mortality and progress towards sustainable development goal target 3.4. Lancet. (2018) 392:1072–88. doi: 10.1016/S0140-6736(18)31992-5, PMID: 30264707

[58] Macdiarmid JI Kyle J Horgan GW Loe J Fyfe C Johnstone A . Sustainable diets for the future: can we contribute to reducing greenhouse gas emissions by eating a healthy diet? Am J Clin Nutr. (2012) 96:632–9. doi: 10.3945/ajcn.112.038729, PMID: 22854399

[59] Green A Nemecek T Walther B Mathys A. Environmental impact, micronutrient adequacy, protein quality, and fatty acid profiles of plant-based beverages compared with cow's milk: a sustainability assessment. Lancet Planet Health. (2022) 6:S8. doi: 10.1016/S2542-5196(22)00270-4

[60] International Standard Organization. ISO 14040:2006. Environmental management — Life cycle assessment — Principles and Framework. Geneva, Switzerland: International Standard Organization (ISO), (2006).

[61] PRé Sustainability. SimaPro LCA software. (2023). Available online at: http://www.pre-sustainability.com/simapro-lca-software

[62] Goedkoop M Heijungs R Huijbregts M de Schryver A Struijs J van Zelm R. ReCiPe 2008. A life cycle impact assessment method which comprises harmonised category indicators at the midpoint and the endpoint level. First edition. Report 1: characterisation.

[63] Swiss Society for Nutrition (SGE) and Federal Food Safety and Veterinary Office (FSVO). Bern, Switzerland: Swiss Food Pyramid.

[64] Ortenzi F McAuliffe GA Leroy F Nordhagen S van Vliet S del Prado A . Can we estimate the impact of small targeted dietary changes on human health and environmental sustainability? Environ. Impact Assess. Rev. (2023) 102:107222. doi: 10.1016/j.eiar.2023.107222

